# Effectiveness of Nurse Led Intervention on Health Related Quality of Life among Children with Sickle Cell Disease in Oman: A Pilot Study

**DOI:** 10.1155/2019/6045214

**Published:** 2019-12-28

**Authors:** Suthan Pandarakutty, Kamala Murali, Judie Arulappan, Deepa Shaji Thomas

**Affiliations:** ^1^Lecturer of Nursing, College of Health Sciences, University of Buraimi (UOB), P. O. Box 890, P.C. 512, Alburaimi, Oman; ^2^Head of the Department of Pediatric Nursing, Rani Meyyammai College of Nursing, Annamalai University, Chidambaram, India; ^3^Assistant Professor/Head of the Department, Department of Maternal and Child Health, College of Nursing, Sultan Qaboos University, P. O. Box 66, Al Khoud, Muscat, Oman; ^4^Lecturer, College of Nursing, Sultan Qaboos University, P. O. Box 66, Al Khoud, Muscat, Oman

## Abstract

*Introduction.* The children with Sickle Cell Disease (SCD) generally have poor Health Related Quality of Life (HRQOL). The study aimed to evaluate the effectiveness of nurse led intervention on HRQOL among children with SCD.

**Methods:**

A total of 30 samples were selected using convenient sampling. Children with SCD and their caregivers completed Pediatric Quality of Life Inventory (PedsQL) SCD-Module version 3.0. The nurse led intervention was given to the study group for 10 consecutive weeks. The control group received the routine medical care. On completion of 10 weeks, the post-test was conducted.

**Results:**

The participants in study group had poor HRQOL scores (*P* > 0.05) in pre-test. After nurse led intervention, the HRQOL score in the study group significantly improved (*P* > 0.05) in pre-test. After nurse led intervention, the HRQOL score in the study group significantly improved (*Discussion.* Therefore nurse led intervention is effective in improving HRQOL among children with SCD.

## 1. Introduction

Sickle Cell Disease (SCD) is the most common inherited blood disorder [1]. Currently millions of people are affected across the globe [2]. More than 250 million people are carrying the gene of SCD and about 25 million people worldwide are living with SCD of which 12–15 million people live in Africa. Worldwide, 257,000 children with sickle cell disease are identified out of 330,000 children born with a major hemoglobinopathy, which makes it the commonest haemoglobin disorder [3].

SCD affects approximately 100,000 Americans and 3 million people have sickle cell trait [1]. SCD commonly occurs among people of African American decent. One in every 365 African Americans and one in every 16,300 Hispanic Americans are born with SCD every year. One in every 13 black or African Americans are born with sickle cell trait [1, 4].

In the Middle Eastern Arab countries, high prevalence of SCD is seen. In Arab countries, the clinical picture of SCD is expressed in benign and severe forms. These forms are related to two distinct *β* globin haplotypes. They are said to be Saudi—Indian and the Benign haplotypes. Modell & Darlison [5] reported that 0.84 of every 1000 live births are affected by HbS (Sickle Haemoglobin) in the Middle East. This accounts for 7389 homozygous neonates per year. In Middle Eastern countries, the prevalence of SCD is very common due to increasing rates of consanguineous marriages, which accounts for more than 50% of marriage between the first cousins [6, 7]. Saudi Arabia's prevalence rate of sickle cell anaemia was estimated at 24 per 10,000. The regional distribution has shown that in the eastern and southern regions the highest prevalence rates are found [8].

It is reported that SCD in Oman is considered to be one of the most common genetic disorders that contributes to increased mortality and morbidity rates in the country [9]. Rajab and Al Salmi [10] reported that there are around 400 patients with Thalassemia major and 3000 patients with sickle cell disorders are identified in Oman. Approximately 6% of Omani's are identified to be the carriers of sickle cell anaemia with the prevalence rate of 1 in 370 or 2.7 per 1000 live births [11]. Rajab, Patton and Modell [12] estimated that in Oman every year 118 new cases are expected to be born with SCD and the projected rate of recurrence is 10%. Regional distribution of SCD revealed a higher prevalence (more than 70% of cases) in regions with smear-positive malaria rates of 1 to >5% (Dhahira, Dakhliya, North and South Shargiya) [12].

**Figure 1:**
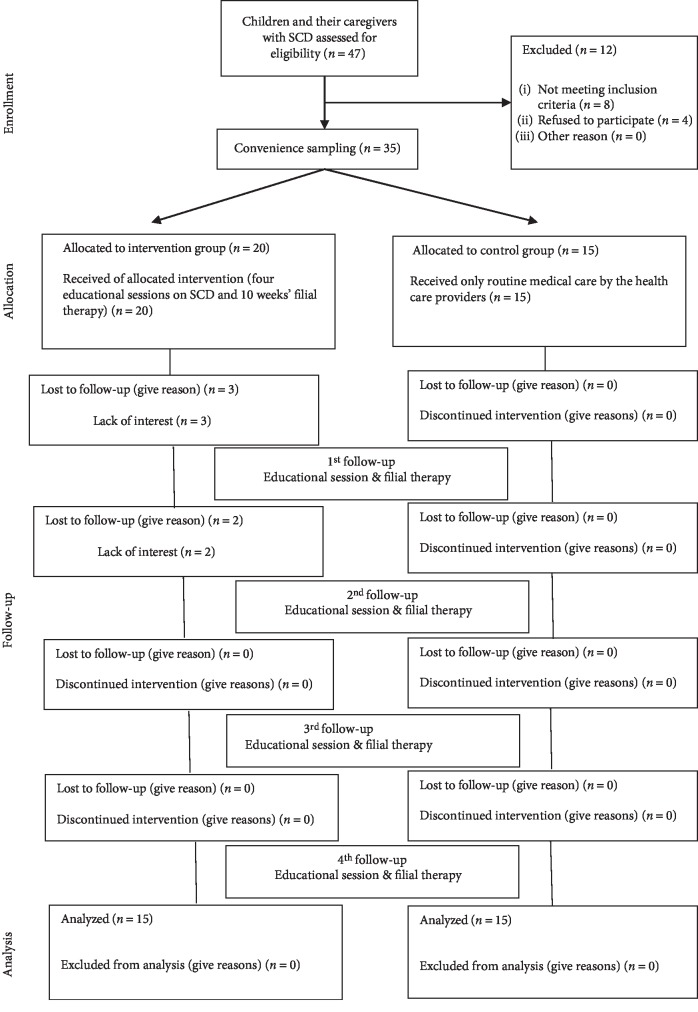
CONSORT diagram of study participants in control and intervention groups [39].

Although SCD treatment has advanced in the last 10 years, the HRQOL (Health Related Quality of life) of children with SCD remains poor [13]. Therefore, the focus should be on improving the HRQOL of children with SCD [14]. Studies shows that children with SCD pose poor quality of life compared to the healthy children of their age due to frequent pain crisis that consequently affects their physical, mental, and psychosocial health [15]. Children, who experienced frequent painful crisis and stroke due to blockage of flow in the brain, had poor memory and attention in the class [16]. SCD severely affects the families of children with SCD. The burden of caregivers increases, family relationships gets disrupted and routine activities gets affected [17]. SCD affects the emotional well-being, school attendance, and school activities of the children with SCD that results in poor HRQOL [18]. The literature states that the presence of SCD in children affects both the families and children with SCD. In Oman, the previous literatures reported that the prevalence of SCD is high. This will have an influence on the HRQOL of children with SCD. Moreover, no interventions have been planned to improve the HRQOL of children with SCD. Therefore, this pilot study is aimed to evaluate the efficacy of a Nurse Led Intervention on HRQOL among Children with Sickle Cell Disease in Oman to assess the feasibility to proceed to a final study with more samples (Figure 1).

## 2. Methods

A quasi-experimental nonequivalent control group design was adopted in the study. The study was conducted in Sultanate of Oman from May 2018 to November 2018. In order to avoid the sample contamination, the intervention and control groups were selected separately from two different regional Ministry of Health hospitals, in Sultanate of Oman. The population comprised of children with SCD aged 8–12 years who attended the paediatric outpatient haematology clinic for regular follow-up medical care that operates on every Tuesdays. Children with SCD who met the inclusion criteria were selected using convenient sampling technique. A total of 35 eligible samples were selected in this study which were assigned to 20 samples in intervention and 15 samples in the control group. However 5 samples in the intervention group dropped out due to lack of interest. Therefore, 15 samples in the intervention and another 15 samples in the control group were included for the analysis.

The inclusion criteria included both male and female children with SCD, aged 8–12 years, who were accompanied by their parents to the paediatric haematology clinic; children who were present during the period of data collection and children with SCD who were able to speak, write, and understand Arabic. Children with other types of hemoglobinopathies such as thalassemia and G6PD, children who were diagnosed to have sickle cell trait, children with SCD who were terminally ill, children with SCD who had undergone splenectomy and children with SCD who were not willing to participate in the study were excluded from the study.

The researcher identified the eligible participants based on the inclusion and exclusion criteria. The researcher approached each participant and distributed the study questionnaire and explained the purpose, benefits, and sought voluntary participation. Participants who agreed to participate were further explained the study procedures and asked to sign in the consent form. After completing the consent process, the participants were requested to assemble in a play room near the paediatric haematology clinic and directed them to complete the pre-test questionnaires, using paper and pen. PedsQL-SCD Module was completed by caregivers and their children.

Data were collected using questionnaires. The data collection questionnaire consisted of two parts. The first part includes 15 questions of demographic variables and 15 questions of clinical variables of children with SCD. The second part of the questionnaire includes Pediatric Quality of Life Inventory (PedsQL) SCD Module [19]. PedsQL-SCD modules of Child Report and the Parent Report was used in the study. The PedsQL-SCD Module had 43 items, and 9 scales: Pain & hurts (9 items), pain impact (10 items), pain management & control (2 items), worry I (5 items), worry II (2 items), emotion (2 items), treatment (7 items), communication I (3 items), and communication II (3 items). The scale is a 5-point Likert scale (0 = never a problem, 1 = almost never a problem, 2 = sometimes a problem, 3 = often a problem, 4 = almost always a problem). Following the instructions for scoring, 0–4 scores were reversely converted to 0–100 scores for standardized interpretation, so that 0 was scored as 100, 1 was scored as 75; 2 was scored as 50; 3 was scored as 25 and 4 was scored as 0. The items were averaged so that the total scores range from 0 to 100; the higher the score, the better paediatric HRQOL [20]. The original PedsQL-SCD tool was available only in English. (0.88 Child Self-Report, 0.90 Parent Proxy-Report) In order to make the participants understand the tool and to get the most accurate data, the tool was translated to Arabic, and was validated by 3 experts in the Arabic language. The tool was then back translated from Arabic to English and the validity and reliability of the tool was tested. The experts rated the tools as a valid tool. The internal consistency reliability score of Arabic tool was (0.82 Child Self-Report; 0.84 Parent Proxy-Report). The nurse led intervention was also validated by experts in the field before conducting the study and the researcher had undergone training and certification courses for fulfilling the requirements in order to ensure interventional fidelity.

**Figure 2:**
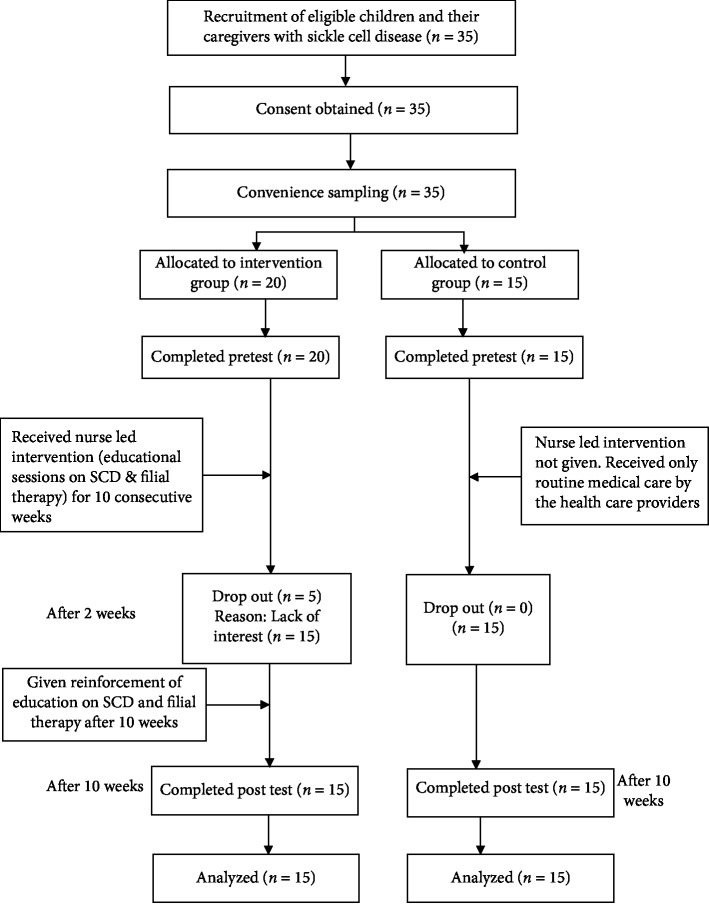
Flow chart of the study data collection and intervention.

In the intervention group, after completion of pre-test, the researcher introduced the study intervention which included educational sessions and filial therapy. A total of four educational sessions on SCD was given for a period of four consecutive weeks to the caregivers and their children with SCD in intervention group. Each educational session lasted for 40–45 minutes. The educational sessions were delivered using PowerPoint Presentation with relevant pictures and videos on SCD. The educational session included information such as basic understanding of SCD, signs and symptoms, complications, diagnosis, medical treatment, homecare management of signs and symptoms, prevention of SCD related complications, coping strategies, emotional, social, and schooling aspects of SCD and available social support system for SCD in Oman. The traditional 10-week filial session [21] was introduced to the caregivers of children with SCD. A total of 10 sessions of filial therapy was given for the period of 10 consecutive weeks. Each filial session lasted for about 1 to 1 and half hours. After completion of 10 sessions of filial therapy the post-test was conducted using the same tool (Figure 2).

In the control group, only pre-test and post-test was conducted and there was no intervention given by the researcher. The control group received only the routine medical care by the health care providers during the scheduled medical appointment with the doctor. After completion of 10 weeks from the pre-test, the post-test was conducted using the same tool.

The data were analysed using descriptive and inferential statistics. The descriptive statistics like frequency, percentage, mean, Mean Difference, and standard deviation were used to interpret the data. Inferential statistics such as Mann-Whitney *U*-test and Wilcoxon signed rank test were used for data analysis. All analyses were conducted using the Statistical Package for the Social Sciences (SPSS) v 24.0.

## 3. Findings

The overall findings from this study suggest that participants in intervention group had poor HRQOL scores (*P* > 0.05) in pre-test than the post-test (^∗^*P* < 0.05). The control group participants had poor HRQOL scores both in the pre-test and post-test (*P* > 0.05). Therefore, the nurse led intervention is found to be effective on HRQOL among children with SCD.  Table 1 shows that there is no significant difference (*P* > 0.05) in all dimensions of HRQOL scores of children with SCD in the study and control group in pre-test.  Table 2 shows that there is a significant difference (^∗^*P* < 0.05) in HRQOL scores of children with SCD in the study and control group in post-test. However, some dimensions of HRQOL scores such as pain impact, emotions and communication I & II did not show any significant improvement.  Table 3 shows that there is a significant difference (^∗^*P* < 0.05) in HRQOL scores of children with SCD in the intervention group in post-test than the pre-test. However, in the parent report, emotions and communication I & II aspects of HRQOL scores did not show any significant improvement. This table also shows that there is no significant difference (*P* > 0.05) in all dimensions of HRQOL scores of children with SCD in the control group in post-test than the pre-test.

## 4. Discussion

In general, the results of the study show that the participants of intervention group had better HRQOL than the control group after the nurse led intervention, which is consistent with findings of other studies [18, 22, 23]. The overall HRQOL of children with SCD in the control group remained poor in terms of pain, worry, emotions, treatment, and communications. These findings are similar in other studies [24–26]. However, the HRQOL of children with SCD in the intervention group significantly improved after the nurse led intervention which shows that the nurse led intervention was effective and therefore, it is one of the recommended interventions for improving HRQOL of children with SCD.

The study revealed that children with SCD had very poor HRQOL score in terms of pain impact, pain crisis and pain management and control in the study and control group before intervention. Therefore, this study highlighted that pain is one of the important predictor for poor HRQOL score in terms of physical, psychological and social functioning which is supported by similar finding of other studies [27–30]. However, the pain impact, pain crisis, pain management and control scores have significantly improved (^∗^*P* < 0.05) after nurse led intervention in intervention group.

Studies have shown that the children with SCD have poor psychological health and lack of family interaction which remains one of the most common problems due to frequent pain crisis, lack of knowledge of the disease and disrupted family relationships [16, 31, 32]. Similar to these study results, this study also identified that the children with SCD had poor psychological function and communication before nurse led intervention. However, after nurse led intervention these scores have significantly improved (^∗^*P* < 0.05). Therefore, nurse led intervention has played an important role in reducing emotions, improving knowledge of the disease and enhancing parental support.

Very few studies have been conducted in Oman regarding HRQOL among children with SCD. In a study conducted in Oman, it is reported that the HRQOL of Omani children with Sickle Cell Anemia remains poor in terms of physical, psychological, emotional, social and school functioning. It is interesting to note that the psychological and emotional domains of HRQOL were severely affected. This study also reported that the haemoglobin F out-weighed white blood cell count which is an important predictor of poor HRQOL in Omani children with SCA [33]. Similar to the study results of Boulassel et al. [33], our study also highlighted that Omani children had poor HRQOL scores in terms of emotional and psychological domains.

Another study conducted in Oman mentioned that the quality of life among children with SCD remains poor mainly due to lack of awareness about the disease. Therefore, health promotion and educational programmes are needed in order to increase the public awareness on SCD and the value of Premarital Screening (PMS) [34]. A study conducted in Oman shows that Hematopoietic stem cell transplantation (HSCT) improves organ function, quality of life, and facilitates disease-free survival [35].

Though, nonnursing interventions supports the improvement of HRQOL of children with SCD, no nurse-led, culturally-tailored, interventional studies have been conducted in Oman in order to improve the HRQOL of Omani children. Therefore, this is the first nurse led interventional study in Oman consisting of educational sessions on SCD and 10 weeks' filial therapy in order to improve the overall HRQOL among children with SCD. The results of this study showed a significant improvement in the overall HRQOL of children with SCD after educational intervention. Findings of this results are supported by other studies [36, 37]. Filial therapy improved the psychological function, parenting, and communication skills which resulted in reduced emotions, strengthened family relationships, and improved social and school functioning. Findings of the study results are similar to other similar studies [38]. However, there are no studies available in Oman regarding the effect of filial therapy on HRQOL among children with SCD.

This pilot study revealed that the nurse-led intervention has significantly improved the overall HRQOL of children with SCD in the intervention group in terms of problem with pain, pain impact, pain management and control, emotions and communications which therefore resulted in enhanced physical, psychological and social functions, enhanced parental support, improved family interactions and improved school performance. However, the results of this pilot study cannot be generalised due to the lower sample size and interventional impact should be observed with the inclusion of higher sample size.

The limitation of the study is that the study included only 30 samples, with which the results of the study cannot be generalized. Another limitation of the study is that there were few drop outs in the study group during the course of the study period which limited the sample size further which is depicted in the CONSORT diagram.

## 5. Conclusion

It is observed that the HRQOL of children with SCD in the intervention group after the nurse led intervention has significantly improved (^∗^*P* < 0.05) than the control group. Therefore, nurse-led intervention is an effective and recommended intervention in improving HRQOL of children with SCD. Education on SCD improves knowledge and the awareness about the disease condition, prevention, and control of SCD-related complications and strengthens coping abilities. Moreover, the filial therapy allows the caregivers to better interact with the child, understand their emotions and improves overall HRQOL of children with SCD. Findings in this study have important implications for proceeding to the final study in a large scale for the complete evaluation of HRQOL of children with SCD in Oman.

## Figures and Tables

**Table 1:** Comparison between pre-test HRQOL Score of participants in the intervention and control group.

Age group	Dimensions of HRQOL	Group				Mean difference	Mann–Whitney *U*-test
		Study		Control			
		Mean	SD	Mean	SD		
8–12 years child report	Pain and hurt	30.67	4.10	29.63	5.74	1.04	*Z* = 1.02
							*P* = 0.30
	Pain impact	26.50	4.98	25.37	4.46	1.13	*Z* = 0.65
							*P* = 0.51
	Pain management and control	29.27	5.14	27.87	8.25	1.40	*Z* = 0.15
							*P* = 0.87
	Worry I	33.13	6.59	31.20	5.48	1.93	*Z* = 0.99
							*P* = 0.31
	Worry II	38.47	7.85	37.03	6.62	1.44	*Z* = 0.08
							*P* = 0.93
	Emotions	38.73	7.97	35.93	6.59	2.80	*Z* = 1.71
							*P* = 0.08
	Treatment	33.81	4.57	31.67	9.04	2.14	*Z* = 0.38
							*P* = 0.70
	Communication I	42.56	4.10	40.64	4.46	1.92	*Z* = 0.67
							*P* = 0.50
	Communication II	25.27	3.05	23.73	2.75	1.54	*Z* = 1.37
							*P* = 0.16

8–12 years parent report	Pain and hurt	29.15	4.49	27.59	7.08	1.56	*Z* = 0.14
							*P* = 0 .88
	Pain impact	27.00	3.68	26.53	9.62	0.47	*Z* = 0.92
							*P* = 0.35
	Pain management and control	28.33	14.54	26.67	13.25	1.66	*Z* = 0.26
							*P* = 0.79
	Worry I	34.00	7.61	33.33	8.34	0.67	*Z* = 0.18
							*P* = 0.85
	Worry II	39.50	12.23	36.67	17.97	2.83	*Z* = 0.43
							*P* = 0.66
	Emotions	36.67	8.80	35.00	11.76	1.67	*Z* = 0.57
							*P* = 0.57
	Treatment	33.65	9.89	30.47	6.92	3.18	*Z* = 1.47
							*P* = 0.13
	Communication I	36.29	7.83	35.20	9.54	1.09	*Z* = 0.25
							*P* = 0.80
	Communication II	28.89	6.95	25.00	9.45	3.89	*Z* = 1.25
							*P* = 0.21

**Table 2:** Comparison between post-test HRQOL score of participants in the intervention and control group.

Age group	Dimensions of HRQOL	Group				Mean difference	Mann–Whitney *U*-test
		Study		Control			
		Mean	SD	Mean	SD		
8–12 years child report	Pain and hurt	40.81	5.46	32.00	8.52	8.81	**Z** = 2.68
							^∗∗^ **P** = 0.01
	Pain impact	32.30	5.08	28.20	7.84	4.1	*Z* = 1.65
							*P* = 0.10
	Pain management and control	43.33	6.13	32.47	6.05	10.86	**Z** = 3.74
							^∗∗∗^ **P** = 0.001
	Worry I	41.93	5.64	34.70	6.20	7.23	**Z** = 2.89
							^∗∗^ **P** = 0.01
	Worry II	44.83	7.77	39.27	6.26	5.56	**Z** = 2.57
							^∗∗^ **P** = 0.01
	Emotions	46.50	7.25	36.33	8.24	10.17	**Z** = 2.88
							^∗∗^ **P** = 0.01
	Treatment	44.70	10.01	34.56	7.00	10.14	**Z** = 2.83
							^∗∗^ **P** = 0.01
	Communication I	50.22	10.19	44.13	14.67	6.09	*Z* = 0.96
							*P* = 0.33
	Communication II	36.93	11.47	27.55	9.36	9.38	*Z* = 1.69
							*P* = 0.09

8–12 years parent report	Pain and hurt	38.19	6.61	29.00	6.20	9.19	**Z** = 3.07
							^∗∗^ **P** = 0.01
	Pain impact	33.37	7.01	27.77	9.57	5.60	*Z* = 1.69
							*P* = 0.09
	Pain management and control	42.97	9.14	29.83	7.76	13.14	**Z** = 3.46
							^∗∗∗^ **P** = 0.001
	Worry I	45.00	9.45	34.20	6.88	10.8	**Z** = 3.44
							^∗∗^ **P** = 0.01
	Worry II	44.00	7.80	37.33	11.05	6.67	**Z** = 1.98
							^∗^ **P** = 0.05
	Emotions	41.67	7.72	38.67	12.42	3.00	*Z* = 0.55
							*P* = 0.58
	Treatment	37.37	7.33	32.08	9.38	5.29	**Z** = 2.10
							^∗^ *P* = 0.04
	Communication I	38.89	6.34	37.51	9.88	1.38	*Z* = 033
							*P* = 0.73
	Communication II	32.53	6.73	28.11	11.11	4.42	*Z* = 1.60
							*P* = 010

^∗^Significant at *P* < = 0.05 , ^∗∗^significant at *P* < = 0.01, ^∗∗∗^significant at *P* < = 0.001.

**Table 3:** Comparison between pre-test and post-test HRQOL score of participants in the intervention and control group.

Age group	Dimensions of HRQOL	Test				Mean difference	Wilcoxon signed rank test	Test				Mean difference	Wilcoxon signed rank test
		Pre-test		Post-test				Pre test		Post test			
		Mean	SD	Mean	SD								
8–12 years child report	Pain and hurt	30.67	4.10	40.81	5.46	10.14	**Z** = 3.42	29.63	5.74	32.00	8.52	2.37	*Z* = 1.02
							^∗∗∗^ **P** = 0.001****						*P* = 0.31
	Pain impact	26.50	4.98	32.30	5.08	5.8	**Z** = 2.44	25.37	4.46	28.20	7.84	2.83	*Z* = 1.14
							^∗^ *P* = 0.02						*P* = 0.25
	Pain management and control	29.27	5.14	43.33	6.13	14.06	**Z** = 3.41	27.87	8.25	32.47	6.05	4.6	*Z* = 1.44
							^∗∗∗^ **P** = 0.001****						*P* = 0.14
	Worry I	33.13	6.59	41.93	5.64	8.8	**Z** = 3.49	31.20	5.48	34.70	6.20	3.5	*Z* = 1.72
							^∗∗∗^ **P** = 0.001						*P* = 0.08
	Worry II	38.47	7.85	44.83	7.77	6.36	**Z** = 2.27	37.03	6.62	39.27	6.26	2.24	Z = 0.59
							^∗^ *P* = 0.02						*P* = 0.55
	Emotions	38.73	7.97	46.50	7.25	7.77	**Z** = 3.47	35.93	6.59	36.33	8.24	0.4	*Z* = 0.03
							^∗∗∗^ **P** = 0.001						*P* = 0.97
	Treatment	33.81	4.57	44.70	10.01	10.89	**Z** = 2.95	31.67	9.04	34.56	7.00	2.89	*Z* = 0.91
							^∗∗^ **P** = 0.01						*P* = 0.36
	Communication I	42.56	4.10	50.22	10.19	7.66	**Z** = 2.44	40.64	4.46	44.13	14.67	3.49	*Z* = 1.04
							^∗^ *P* = 0.02						*P* = 0.30
	Communication II	25.27	3.05	36.93	11.47	11.66	**Z** = 2.86	23.73	2.75	27.55	9.36	3.82	*Z* = 1.80
							^∗∗^ **P** = 0.01						*P* = 0.07

8–12 years parent report	Pain and hurt	29.15	4.49	38.19	6.61	9.04	**Z** = 3.25	27.59	7.08	29.00	6.20	1.41	*Z* = 1.00
							^∗∗∗^ **P** = 0.001****						*P* = 0.32
	Pain impact	27.00	3.68	33.37	7.01	6.37	**Z** = 256	26.53	9.62	27.77	9.57	1.24	*Z* = 1.04
							^∗∗^ **P** = 0.01						*P* = 0.31
	Pain management and control	28.33	14.54	42.97	9.14	14.64	**Z** = 2.90	26.67	13.25	29.83	7.76	3.16	*Z* = 0.97
							^^∗∗∗^^ **P** = 0.001****						*P* = 0.33
	Worry I	34.00	7.61	45.00	9.45	11.00	**Z** = 2.37	33.33	8.34	34.20	6.88	0.87	*Z* = 1.06
							^^∗^^ *P* = 0.02						*P* = 0.28
	Worry II	39.50	12.23	44.00	7.80	4.50	*Z* = 1.05	36.67	17.97	37.33	11.05	0.66	*Z* = 0.44
							*P* = 0.31						*P* = 0.66
	Emotions	36.67	8.80	41.67	7.72	5.00	**Z** = 2.12	35.00	11.76	38.67	12.42	3.67	*Z* = 1.34
							^^∗^^ *P* = 0.03						*P* = 0.18
	Treatment	33.65	9.89	37.37	7.33	3.72	**Z** = 2.02	30.47	6.92	32.08	9.38	1.61	*Z* = 1.01
							^^∗^^ *P* = 0.04						*P* = 0.31
	Communication I	36.29	7.83	38.89	6.34	2.60	**Z** = 2.03	35.20	9.54	37.51	9.88	2.31	*Z* = 1.04
							^^∗^^ *P* = 0.04						*P* = 0.31
	Communication II	28.89	6.95	32.53	6.73	3.64	*Z* = 1.86	25.00	9.45	28.11	11.11	3.11	*Z* = 1.39
							*P* = 0.06						*P* = 0.17

^∗^Significant at *P* < = 0.05, ^∗∗^significant at*P* < = 0.01 , ^∗∗∗^significant at *P* < = 0.001.

## Data Availability

The data will be available upon request.
